# Mortality Attributable to Low Levels of Education in the United States

**DOI:** 10.1371/journal.pone.0131809

**Published:** 2015-07-08

**Authors:** Patrick M. Krueger, Melanie K. Tran, Robert A. Hummer, Virginia W. Chang

**Affiliations:** 1 Department of Health & Behavioral Sciences, University of Colorado Denver | Anschutz Medical Campus, Denver, CO, United States of America; 2 Population Program, Institute of Behavioral Sciences, University of Colorado Boulder, Boulder, CO, United States of America; 3 Department of Sociology and Carolina Population Center, University of North Carolina at Chapel Hill, Chapel Hill, NC, United States of America; 4 Steinhardt School of Culture, Education, and Human Development, Global Institute of Public Health, and School of Medicine, New York University, New York, NY, United States of America; Leibniz Institute for Prevention Research and Epidemiology (BIPS), GERMANY

## Abstract

**Background:**

Educational disparities in U.S. adult mortality are large and have widened across birth cohorts. We consider three policy relevant scenarios and estimate the mortality attributable to: (1) individuals having less than a high school degree rather than a high school degree, (2) individuals having some college rather than a baccalaureate degree, and (3) individuals having anything less than a baccalaureate degree rather than a baccalaureate degree, using educational disparities specific to the 1925, 1935, and 1945 cohorts.

**Methods:**

We use the National Health Interview Survey data (1986–2004) linked to prospective mortality through 2006 (N=1,008,949), and discrete-time survival models, to estimate education- and cohort-specific mortality rates. We use those mortality rates and data on the 2010 U.S. population from the American Community Survey, to calculate annual attributable mortality estimates.

**Results:**

If adults aged 25–85 in the 2010 U.S. population experienced the educational disparities in mortality observed in the 1945 cohort, 145,243 deaths could be attributed to individuals having less than a high school degree rather than a high school degree, 110,068 deaths could be attributed to individuals having some college rather than a baccalaureate degree, and 554,525 deaths could be attributed to individuals having anything less than a baccalaureate degree rather than a baccalaureate degree. Widening educational disparities between the 1925 and 1945 cohorts result in a doubling of attributable mortality. Mortality attributable to having less than a high school degree is proportionally similar among women and men and among non-Hispanic blacks and whites, and is greater for cardiovascular disease than for cancer.

**Conclusions:**

Mortality attributable to low education is comparable in magnitude to mortality attributable to individuals being current rather than former smokers. Existing research suggests that a substantial part of the association between education and mortality is causal. Thus, policies that increase education could significantly reduce adult mortality.

## Introduction

Low educational attainment is a strong predictor of premature adult mortality in the U.S. and many other countries [1–5]. In the U.S., educational disparities in mortality have been increasing among more recent birth cohorts, with greater improvements in survival accruing to those with more education [6]. Moreover, life expectancies have stagnated among those with less than a high school education, and may even be declining among women with the least education [7–9].

Surprisingly, the number of deaths attributable to low education in the U.S. is not well documented. Most studies on this topic examine the relative risk of death across levels of educational attainment. However, estimates of *attributable mortality* are critical to public health and policy initiatives because they quantify the population-level impact of educational disparities by considering both the relative risk of death across educational attainment and the distribution of education in the population. Three studies estimate the mortality attributable to low education [10–12], but they fail to consider widening educational disparities in mortality across birth cohorts, examine broad educational groups that obscure practical and policy-relevant steps in educational attainment, do not adjust for gender or race, ignore cause of death, or use educational attainment as recorded on death certificates, which is routinely misreported [13].

We estimate excess mortality attributable to US adults aged 25–85 who: (1) have less than a high school degree rather than a high school degree or high school equivalency credential (GED), (2) have some college rather than a baccalaureate degree, and (3) have any level of education that is less than a baccalaureate degree rather than a baccalaureate degree. Maximizing high school graduation rates and the completion of baccalaureate degrees among those who have already entered college are viable policy targets [14]. Evidence suggests that increases at lower levels of the educational distribution are causally linked to reductions in adult mortality [15–17]. Further, a substantial share of adults are exposed; of those aged 25–34 in 2012, 10.7% have not completed a high school degree or GED, and 28.5% have some college but not a baccalaureate degree [18]. We compare attributable mortality based on educational disparities specific to the 1925, 1935, and 1945 birth cohorts to assess the impact of widening disparities over time.

## Methods

We use the 1986–2004 waves of the National Health Interview Survey (NHIS), linked to prospective mortality through 2006 in the Linked Mortality File (LMF), to estimate mortality rates by educational attainment and birth cohort [19]. The NHIS offers annual, cross-sectional, nationally representative surveys of non-institutionalized adults aged 18 and older and has a response rate of 87% or higher for sampled households. The LMF includes deaths from the National Death Index, Social Security Administration, and the Center for Medicare and Medicaid Services, which are probabilistically matched to individuals in the NHIS [20]. About 5.6% of respondents in the NHIS cannot be linked to vital status due to missing information on the matching criteria; following National Center for Health Statistics recommendations, we exclude them from our analyses and use survey weights to adjust for their exclusion.

We exclude individuals younger than age 25 at baseline because many are still enrolled in school, and we exclude adults aged 85 and older because their ages are top-coded. We exclude foreign-born adults between 1989 and 2004 because the NHIS-LMF does not indicate where they received their education and because the National Death Index cannot capture deaths among migrants returning to their countries of origin. The NHIS does not ascertain nativity between 1986 and 1988, so we cannot drop foreign born adults in those waves; the share of the U.S. adult population that was foreign born in those years was just 2.5% [21]. Our final sample includes 468,725 males and 540,224 females, of whom 78,674 and 77,016, respectively, died over the follow-up period.

### Variables

Our outcomes include all-cause mortality, cardiovascular disease mortality (ICD-10 codes I00 through I99) and cancer mortality (ICD-10 codes C00 through C97). We use broad cause-of-death categories to limit the impact of misclassification of underlying causes of death on death certificates. Age at baseline is measured in quarter-years (range 25.0 to 84.75). Birth cohort is also measured in quarter-years (range 1901.25 to 1980.5). Because we use 19 cross-sectional waves of the NHIS, follow vital status for up to 21 years, and allow age to increase throughout the follow-up period, the age and birth cohort variables are not perfectly collinear.

Educational attainment is coded categorically as less than a high school degree, high school degree or GED, some college but no baccalaureate degree, baccalaureate degree, and any post-baccalaureate education. Our variable captures key educational milestones. The NHIS ascertains educational attainment as years completed prior to 1997 and as highest degree completed in 1997 and later; we recode years of education completed in the 1986–1996 waves of data to be consistent with the highest degree measure used in later waves. This coding strategy has been used previously to estimate educational disparities among U.S. adults [6, 7]. Race/ethnicity is coded categorically as non-Hispanic white, non-Hispanic black, non-Hispanic Asian/Pacific Islander, non-Hispanic Native American, Mexican American, Puerto Rican, Cuban, and all others. Sex is measured dichotomously.

### Mortality Rates

We use a complementary log-log discrete time survival model to estimate mortality rates [22]. We create a person-period data set where each person contributes an observation for each quarter-year that they are observed between the date of interview and the date of death (if they died) or the date of censor (the fourth quarter of 2006, if they survived the follow-up period). We use age as the time to event [23]. Age is time-varying in the person-period data. Although we drop those aged 85 and older at baseline, we allow adults who survive past age 85 over the follow-up period to continue contributing to the hazard of death. We compare models that raised age to all combinations of two of the following exponents: 0.5, 1, 1.5, 2, 2.5, and 3 [24]. Raising age to the exponents of 2.5 and 3.0 best fit the data and recovered the patterns observed when using 5-year dummy variables for age (not shown). We include birth cohort as a continuous variable; we found no nonlinear associations between birth cohort and mortality risk.

To allow for widening mortality disparities by educational attainment over time, we include multiplicative interaction terms between the birth cohort and educational attainment variables. We subtract 1925 from the birth cohort variable so that the main effects of the education variables indicate educational disparities in mortality for members of the 1925 birth cohort. Our analyses focus on mortality rates specific to the 1925, 1935, and 1945 birth cohorts; these cohorts contribute deaths between ages 41 and 81 over the follow-up period and allow us to illustrate changes in attributable mortality as educational disparities in mortality have widened in the U.S. None of the three birth cohorts contributes age-specific mortality rates for all ages between 25 and 85, but each contributes age-specific deaths for 21 years over the follow-up period. Consistent with earlier work [6], our models assume that educational disparities for specific cohorts remain constant even at ages that are not observed in our data, allowing for the estimation of mortality rates in each of the cohorts for ages 25–85. Thus, our mortality rates incorporate both period and cohort variation [25]. We avoid consideration of more recent cohorts because those adults are young, contribute relatively few deaths, and their educational disparities in mortality may be estimated imprecisely. We use Stata statistical software to multiply impute the 1.1% of cases that are missing on educational attainment (the only variable with missing data) and to estimate our survival models [26]. (The S1 Appendix shows life tables calculated from our estimated mortality rates to confirm the reasonableness of those rates.)

### Mortality Attributable to Low Educational Attainment

To estimate mortality attributable to low educational attainment, we first calculate the number of deaths we would expect when multiplying the estimated mortality rates (age- and sex-specific and race-adjusted mortality rates, by educational attainment for the 1925, 1935, and 1945 birth cohorts), with the distributions of educational attainment, by age and sex, in the 2010 U.S. population. The 2010 population provides a current distribution of education among U.S. adults, which we use to consider the impact of widening educational disparities in mortality on attributable mortality. We estimate the 2010 U.S. population from the American Community Survey (ACS) [27]. We pool the 2009, 2010, and 2011 waves of the ACS to ensure stable estimates by education, age, sex, and race, and weight the numbers of people to the population enumerated by the 2010 Census. We exclude foreign-born and institutionalized adults from the ACS, just as we do with the NHIS-LMF. Next, we subtract the estimated number of deaths when giving members of some educational groups the lower mortality rates of those with higher levels of education.

We examine three scenarios. Scenario 1 estimates mortality attributable to individuals not completing high school rather than completing high school or a GED. Scenario 2 estimates mortality attributable to individuals not completing a baccalaureate degree among those who have matriculated to college but have not completed a baccalaureate degree. Scenario 3 estimates mortality attributable to individuals having any level of education that is less than a baccalaureate degree rather than a baccalaureate degree. We estimate all models separately by sex given sex differences in the association between education and survival [7]. Additional analyses separately estimate attributable mortality due to low education from cardiovascular disease and cancer deaths, as well as all-cause attributable mortality for whites and blacks (sample sizes for other race/ethnic groups are too small to provide stable estimates). All analyses include sample weights and account for the complex sampling frame by treating strata in subsequent sampling frames as though they come from different (i.e., more recent) populations [28].

### Ethical Approval

This study was approved by the institutional review board at the University of Colorado Denver, and we have conformed to the principles embodied in the Declaration of Helsinki.

## Results

Table 1 presents means and percentages of the study variables from the NHIS-LMF by sex and vital status. Individuals who are older, born in earlier birth cohorts, and who have lower levels of educational attainment are more likely to have died during the follow-up period than their counterparts.

**Table 1:** Means (Standard Deviations) and Percentages of Study Variables, By Sex and By Vital Status at the End of the Follow-Up Period, Non-Institutionalized U.S. Born Adults Aged 25 and Older.

	Men			Women		
	Survived	Died	p-value^a^	Survived	Died	p-value^a^
Age, mean	52.0	71.8	<0.001	53.8	75.7	<0.001
	(14.5)	(13.5)		(15.6)	(13.1)	
Birth Cohort (years), mean	1947.6	1928.2	<0.001	1945.9	1924.8	<0.001
	(14.7)	(13.8)		(15.7)	(13.2)	
Educational Attainment, %						
No high school degree	16.8	37.1	<0.001	17.3	39.3	<0.001
High school degree or GED	35.3	32.8		39.4	37.2	
Some college	21.6	15.5		22.7	14.4	
Baccalaureate degree	15.1	8.1		12.7	5.6	
Any post-baccalaureate	11.3	6.5		7.8	3.4	
Race/Ethnicity, %						
White	83.0	83.9	<0.001	81.3	83.9	<0.001
Black	10.0	11.4		11.7	11.8	
Asian	1.0	0.5		1.0	0.5	
Mexican American	2.8	1.8		2.7	1.5	
Native American	0.7	0.7		0.7	0.6	
Puerto Rican	0.5	0.3		0.6	0.3	
Cuban	0.3	0.2		0.3	0.2	
Other	1.7	1.2		1.8	1.3	
Survival, %						
Overall mortality	0.0	100.0		0.0	100.0	
Cancer		27.1			24.4	
Cardiovascular Disease		38.5			39.4	
N (person)	390,051	78,674		463,208	77,016	
N (person quarters)	21,017,629	78,674		24,858,129	77,016	

Source: National Health Interview Survey-Linked Mortality File, 1986–2006.

^a^P-values indicate whether the distributions of study variables are significantly different for those who survived and those who died over the follow-up period, by sex.

Table 2 presents hazard ratios from survival models and shows that among both males and females, educational attainment has a graded, inverse association with the risk of death. For example, compared to males with a high school degree or GED in the 1925 cohort (when cohort equals zero), males with no high school degree have a 23% higher risk of death, males with some college have a 6% lower risk of death, males with baccalaureate degrees have a 25% lower risk of death, and males with post-baccalaureate education have a 33% lower risk of death. The hazard ratio for birth cohort shows that the risk of death among males with a high school degree or GED (the referent group for educational attainment) falls by 1% in each subsequent 1-year birth cohort. Among females with a high school degree or GED, the risk of death does not vary by birth cohort. The education by birth cohort interaction terms for both men and women show that the risk of death in each subsequent cohort falls more rapidly among those with more education.

**Table 2:** Hazard Ratios and 95% Confidence Intervals from Survival Models for Overall Mortality, Non-Institutionalized U.S. Born Adults Aged 25 and Older.

	Males	Females
(Age)^2.5^	1.00022***	1.00022***
	(1.00021,1.00023)	(1.00021,1.00024)
(Age)^3.0^	0.999984***	0.999984***
	(0.999983,0.999984)	(0.999983,0.999985)
Educational Attainment		
No high school degree	1.23***	1.32***
	(1.21,1.25)	(1.29,1.34)
High school degree or GED	ref.	ref.
Some college	0.94***	0.92***
	(0.91,0.96)	(0.90,0.95)
Baccalaureate degree	0.75***	0.78***
	(0.73,0.77)	(0.76,0.81)
Any post-baccalaureate	0.67***	0.75***
	(0.65,0.70)	(0.72,0.78)
Birth Cohort	0.990***	1.001
	(0.988,0.992)	(0.999,1.003)
Interactions: Date of birth by		
No high school degree	1.011***	1.015***
	(1.010,1.013)	(1.01,1.02)
High school degree or GED	ref.	ref.
Some college	0.998	0.997***
	(0.997,1.000)	(0.995,0.998)
Baccalaureate degree	0.988***	0.987***
	(0.986,0.991)	(0.985,0.990)
Post-baccalaureate education	0.983***	0.984***
	(0.981,0.986)	(0.981,0.987)
Race/Ethnicity		
White	ref.	ref.
Black	1.26***	1.21***
	(1.23,1.30)	(1.17,1.24)
Asian	0.64***	0.64***
	(0.58,0.71)	(0.56,0.73)
Mexican American	0.83***	0.79***
	(0.79,0.88)	(0.73,0.86)
Native American	1.13*	1.14*
	(1.01,1.27)	(1.01,1.29)
Puerto Rican	0.94	0.96
	(0.78,1.13)	(0.80,1.14)
Cuban	0.84*	0.88*
	(0.73,0.98)	(0.79,1.00)
Other	0.84***	0.86***
	(0.78,0.90)	(0.79,0.94)
Intercept	0.001***	0.00039***
	(0.0009,0.0011)	(0.00035,0.00043)

Source: National Health Interview Survey-Linked Mortality File, 1986–2006.

* p < .05;

** p < .01;

*** p < .001.

Table 3 shows the annual mortality attributable to low educational attainment. Results for Scenario 1 show that the annual mortality attributable to individuals having less than a high school degree, rather than a high school degree or GED, is 68,512 (9% of adult deaths in the 25–85 age range) among males and 76,731 (12% of adult deaths in the 25–85 age range) among females, based on educational disparities in mortality in the 1945 birth cohort. The share of deaths attributable to individuals having less than a high school degree increases more quickly across cohorts for females than for males due to the increasing mortality rates among low-educated females over time (see Table 2). Results for Scenario 2 show that the annual mortality attributable to individuals having some college, rather than a baccalaureate degree, is 68,293 (9% of adult deaths in the 25–85 age range) among males and 41,776 (7% of adult deaths in the 25–85 age range) among females, based on educational disparities in mortality in the 1945 birth cohort. Results for Scenario 3 show that the annual mortality attributable to individuals having anything less than a baccalaureate degree, rather than a baccalaureate degree, is 293,823 (37% of adult deaths in the 25–85 age range) among males, and 251,702 (39% of adult deaths in the 25–85 age range) among females, based on educational disparities in the 1945 birth cohort. Results for all three scenarios show that the annual attributable mortality nearly doubles due to widening educational disparities in mortality between the 1925 and 1945 birth cohorts.

**Table 3:** Annual Mortality Attributable to Low Education, Numbers and Percentages of Deaths, by Sex and Birth Cohort, Non-Institutionalized U.S. Born Adults Aged 25 to 85 in the 2010 Population.

		Men		Women		Total	
		Number	% of total	Number	% of total	Number	% of total
**Scenario 1:** Mortality attributable to having less than a high school degree rather than a high school degree or GED.							
Birth Cohort	1945	68,512	9%	76,731	12%	145,243	10%
	1935	52,916	6%	52,537	8%	105,453	7%
	1925	35,958	4%	31,714	5%	67,672	4%
**Scenario 2:** Mortality attributable to having some college rather than a baccalaureate degree							
Birth Cohort	1945	68,293	9%	41,776	7%	110,068	8%
	1935	61,419	7%	32,930	5%	94,350	6%
	1925	50,092	5%	22,618	4%	72,710	5%
**Scenario 3:** Mortality attributable to having less than a baccalaureate degree rather than a baccalaureate degree							
Birth Cohort	1945	293,823	37%	251,702	39%	545,525	38%
	1935	255,970	29%	189,897	30%	445,867	30%
	1925	203,281	21%	126,811	21%	330,092	21%

Source: National Health Interview Survey-Linked Mortality File, 1986–2006; American Community Survey 2009 to 2011.

Note: All estimates are adjusted for race/ethnicity.

Table 4 shows that the percentage of deaths attributable to low education is greater for cardiovascular disease than for cancer. Based on educational disparities in mortality in the 1945 birth cohort, 16% of cardiovascular disease deaths among females and 9% of cardiovascular disease deaths among males are attributable to individuals having less than a high school degree rather than a high school degree or GED. In contrast, based on educational disparities in mortality in the 1945 birth cohort, 7% of cancer deaths among females and males are attributable to individuals having less than a high school degree rather than a high school degree.

**Table 4:** Annual Cause Specific Mortality Attributable to Low Education, Numbers and Percentages of Deaths, by Sex and Birth Cohort, Non-Institutionalized U.S. Born Adults Aged 25 to 85 in the 2010 Population.

		Men		Women		Total	
		Number	% of total	Number	% of total	Number	% of total
**Panel A: Cardiovascular Disease Mortality**							
**Scenario 1:** Mortality attributable to having less than a high school degree rather than a high school degree or GED.							
Birth Cohort	1945	18,246	9%	22,449	16%	40,695	12%
	1935	16,460	6%	18,683	11%	35,143	8%
	1925	13,196	4%	13,880	7%	27,076	5%
**Scenario 2:** Mortality attributable to having some college rather than a baccalaureate degree							
Birth Cohort	1945	18,360	9%	10,062	7%	28,422	8%
	1935	18,710	7%	9,989	6%	28,699	7%
	1925	16,902	5%	8,783	4%	25,685	5%
**Scenario 3:** Mortality attributable to having less than a baccalaureate degree rather than a baccalaureate degree							
Birth Cohort	1945	81,669	39%	63,920	46%	145,589	42%
	1935	82,707	31%	59,525	36%	142,232	33%
	1925	76,353	22%	49,806	25%	126,159	23%
**Panel B: Cancer Mortality**							
**Scenario 1:** Mortality attributable to having less than a high school degree rather than a high school degree or GED.							
Birth Cohort	1945	15,087	7%	12,223	7%	27,311	7%
	1935	11,797	5%	7,728	4%	19,526	5%
	1925	8,146	3%	3,500	2%	11,646	3%
**Scenario 2:** Mortality attributable to having some college rather than a baccalaureate degree							
Birth Cohort	1945	13,941	7%	9,752	5%	23,694	6%
	1935	14,049	6%	6,847	4%	20,896	5%
	1925	13,678	5%	3,150	2%	16,828	4%
**Scenario 3:** Mortality attributable to having less than a baccalaureate degree rather than a baccalaureate degree							
Birth Cohort	1945	66,198	32%	48,584	27%	114,782	30%
	1935	58,763	25%	34,048	18%	92,810	22%
	1925	48,246	18%	17,338	9%	65,584	14%

Source: National Health Interview Survey-Linked Mortality File, 1986–2006; American Community Survey 2009 to 2011.

Note: All estimates are adjusted for race/ethnicity.

Table 5 shows that the percentage of deaths attributable to low education is similar for non-Hispanic whites and non-Hispanic blacks given educational disparities in mortality in the 1945 birth cohort, and that mortality attributable to low education is increasing across cohorts for both groups. Mortality attributable to individuals having some college rather than a baccalaureate degree, and to individuals having anything less than a baccalaureate degree rather than a baccalaureate degree, is increasing more quickly across cohorts for blacks than for whites. For example, 5% of deaths among whites but just 1% of deaths among blacks are attributable to having some college rather than a baccalaureate degree, given educational disparities in mortality in the 1925 cohort. Given educational disparities in the 1945 birth cohort, however, the percentage of deaths attributable to having some college rather than a baccalaureate degree is more similar, comprising 7% of deaths among whites and 6% of deaths among blacks.

**Table 5:** Annual Race-Specific Mortality Attributable to Low Education, Numbers and Percentages of Deaths, by Sex and Birth Cohort, Non-Institutionalized U.S. Born Adults Aged 25 to 85 in the 2010 Population.

		Men		Women		Total	
		Number	% of total	Number	% of total	Number	% of total
**Panel A: Non-Hispanic Whites**							
**Scenario 1:** Mortality attributable to having less than a high school degree rather than a high school degree or GED.							
Birth Cohort	1945	46,744	8%	61,073	12%	107,817	10%
	1935	37,016	6%	41,516	8%	78,532	7%
	1925	26,494	4%	24,914	5%	51,408	4%
**Scenario 2:** Mortality attributable to having some college rather than a baccalaureate degree							
Birth Cohort	1945	50,030	9%	32,539	6%	82,569	7%
	1935	45,236	7%	26,182	5%	71,419	6%
	1925	37,442	5%	18,899	4%	56,341	5%
**Scenario 3:** Mortality attributable to having less than a baccalaureate degree rather than a baccalaureate degree							
Birth Cohort	1945	207,154	36%	200,682	38%	407,836	37%
	1935	182,151	28%	152,926	30%	335,077	29%
	1925	147,502	21%	105,204	21%	252,707	21%
**Panel B: Non-Hispanic Blacks**							
**Scenario 1:** Mortality attributable to having less than a high school degree rather than a high school degree or GED.							
Birth Cohort	1945	9,951	10%	11,159	12%	21,111	11%
	1935	7,502	7%	8,269	9%	15,771	8%
	1925	4,710	4%	5,545	6%	10,256	5%
**Scenario 2:** Mortality attributable to having some college rather than a baccalaureate degree							
Birth Cohort	1945	5,686	6%	6,153	7%	11,839	6%
	1935	3,545	3%	4,314	5%	7,859	4%
	1925	385	0.3%	2,035	2%	2,421	1%
**Scenario 3:** Mortality attributable to having less than a baccalaureate degree rather than a baccalaureate degree							
Birth Cohort	1945	31,723	32%	35,083	38%	66,805	35%
	1935	22,935	21%	25,588	28%	48,523	24%
	1925	10,622	9%	14,850	16%	25,472	12%

Source: National Health Interview Survey-Linked Mortality File, 1986–2006; American Community Survey 2009 to 2011.

Note: All estimates are adjusted for race/ethnicity.

## Discussion

Higher educational attainment is inversely associated with U.S. adult mortality through mechanisms including higher income and social status, enhanced cognitive development, better adherence to medical treatments, healthier behaviors, and improved social connections and psychological wellbeing [15, 18, 29–31]. Evidence from cohort studies that adjust for numerous confounders (e.g., intelligence, early life conditions) and from natural experiments consistently show strong associations between educational attainment and mortality, which suggests that a substantial part of the association between education and mortality is causal [15–17]. This may especially be true at lower levels of the educational distribution; some research finds a stronger inverse association between educational attainment and adult mortality after accounting for the nonrandom selection of individuals into higher levels of education [17], suggesting that individuals who are less likely to select into additional education benefit more than those who are already likely to earn high school or college degrees. Thus, Healthy People 2020 sets targets for increasing the proportion of students who graduate with regular diplomas four years after starting 9^th^ grade, and for increasing the proportion of adults aged 18–24 who complete a high school education [14].

Our findings suggest that meeting the Healthy People 2020 goals could have a substantial impact on future U.S. survival patterns. The estimated 145,243 deaths in 2010 attributable to individuals having less than a high school degree rather than a high school degree or GED, based on educational disparities in mortality in the 1945 birth cohort, is comparable to the estimated number of deaths that could be averted if all current smokers had the mortality rates of former smokers [32]. Our estimates of the mortality attributable to individuals having less than a high school degree are smaller than the 245,000 deaths estimated by Galea et al. [11] because they combine those with high school degrees with those who have a college education, which overstates the survival benefits of simply finishing high school. Woolf et al. [10] estimate that a more modest 71,000 deaths are attributed to individuals having less than a high school degree, but they use educational attainment from death certificates which is routinely misreported [10]. Jemal et al.[12] do not present findings for mortality attributable to individuals having less than a high school degree.

We estimate that completion of baccalaureate degrees among those with some college education is associated with 110,068 fewer deaths per year in the 25–85 age range, given educational disparities in mortality in the 1945 birth cohort. Some adults who would benefit from a baccalaureate degree may not complete college due to limited economic resources or because they are unaware of the benefits for both health and earnings [15]. We also find that 545,525 deaths per year among adults aged 25–85 are attributable to individuals having anything less than a baccalaureate degree rather than a baccalaureate degree. This upper-bound estimate may be implausible if individuals who have not enrolled in college lack adequate academic preparation or have little interest in pursuing a college degree—even if they might receive health and economic benefits from completing a baccalaureate degree.

The magnitude of widening educational disparities in mortality is substantial, although prior studies of attributable mortality have ignored those trends [10–12]. Increasing educational disparities in mortality across cohorts result from a variety of changes disproportionately benefitting those with more education, including improved labor market outcomes, reduced incidence of medical conditions, better outcomes among those with acute and chronic disease, and greater reductions in smoking [30, 33, 34]. The percentage of all-cause mortality attributable to having less than a high school degree rather than a high school degree or GED doubles among both men and women when considering educational disparities in mortality in the 1945 cohort rather than the 1925 cohort. Maximizing high school graduation rates are especially crucial given the falling life expectancies among women with the least education [7]. Deaths from cardiovascular disease play a greater role than deaths from cancer in widening educational disparities in mortality, likely due to greater improvements in the prevention and treatment of cardiovascular disease over time among those with more education [30]. The share of all-cause mortality attributable to individuals having some college rather than a baccalaureate degree increases more rapidly among blacks than among whites across cohorts, reflecting faster declines in all-cause mortality among blacks with baccalaureate degrees rather than some college, than among whites [18, 33].

There are limitations of our analyses. As with all attributable mortality calculations, including those for other exposures such as smoking, our estimates may be inflated because our data lack information on all confounders that could potentially influence the exposure-mortality association. Potential confounders include childhood health, cognitive ability, genetic predispositions, and childhood socioeconomic conditions such as living with low-educated, unmarried, or impoverished parents in early life. At present, no available data allow us to incorporate all potential confounders. Further, the association between educational attainment and U.S. adult mortality has been robust to inclusion of such confounders when available, suggesting that their inclusion would not fundamentally alter our findings [15, 35, 36]. Our data allow us to adjust for race/ethnicity, stratify by gender, estimate the association between education and mortality across birth cohorts, and ascertain education at baseline rather than from death certificates—characteristics that allow us to advance beyond prior estimates of mortality attributable to low educational attainment. Alternately, our estimates of attributable mortality may be conservative because we exclude deaths among those who are older than age 85, foreign born, or who live in institutions. Finally, our measure of educational attainment does not account for variation in the quality of schooling across time and geographic areas.

Given that over 10 percent of U.S. adults aged 25–34 have less than a high school degree or GED, and 28.5% have some college but not a baccalaureate degree, there remains much room for improvement with regard to U.S. educational attainment [18]. Our results suggest that policies and interventions that improve educational attainment could substantially improve survival in the U.S. population, especially given widening educational disparities across birth cohorts. The magnitude of our estimates confirms the importance of considering education policy as a key element of US health policy [37, 38], and a major concern for current and future physicians [39]. Medical and policy research will benefit from renewed attention to the population-level impact of educational disparities in mortality and the potential for even greater survival inequality in the future.

## Supporting Information

S1 Appendix Life Tables Calculated from our Estimated Mortality Rates. (PDF)
