# Supplementary material for: Mortality Attributable to Low Levels of Education in the United States
Source: PLoS One. 2015 Jul 8;10(7):e0131809. doi: 10.1371/journal.pone.0131809 (PMC4496052; doi:10.1371/journal.pone.0131809)
Supplement: S1 Appendix — (PDF) [file pone.0131809.s001.pdf]

S1 Appendix: Life Tables Calculated from our Estimated Mortality Rates

To confirm that the estimated mortality rates from the survival models are reasonable, we calculate life expectancies for men and women at each educational level, while setting race/ethnicity to the percentage distribution observed in the NHIS-LMF data [1, 2]. SI Table 1 presents estimated life expectancies for the 1925, 1935, and 1945 cohorts, by educational attainment, calculated from the hazard models shown in Table 2. Among males with less than a high school degree, life expectancy changes little across birth cohorts. Among females with less than a high school degree, life expectancy declines across cohorts. Among males with a high school degree or more education, life expectancy increases across cohorts. Among females, life expectancy does not change across cohorts for those who have high school degrees, but increases across cohorts for those with some college or more education. Notably, our estimated life expectancies, for each cohort and by educational attainment, are similar to those estimated from other data and with different methods [3, 4].

References

1. Rogers RG, Hummer RA, Krueger PM, Pampel FC. Mortality attributable to cigarette smoking in the United States. Population and Development Review. 2005;31:259-92.
2. Preston SH, Heuveline P, Guillot M. Demography: measuring and modeling population processes. Malden, MA: Blackwell; 2001.
3. Rostron BL, Boies JL, Arias E. Education reporting and classification on death certificates in the United States: US Department of Health and Human Services, Centers for Disease Control and Prevention, National Center for Health Statistics; 2010.
4. National Center for Health Statistics. Health, United States, 2011: With special feature on socioeconomic status and health. Hayattsville, MD: U.S. Department of Health and Human Services; 2012.

SI Table 1: Life Expectancy Estimates at Selected Ages, by Sex and Educational Attainment, Based on Educational Disparities in Mortality in the 1925, 1935, and 1945 Birth Cohorts, Non-Institutionalized U.S. Born Adults Aged 25 and Older

	No High School Degree			High School Degree or GED			Some College			Baccalaureate Degree			Any Post-Baccalaureate Education		
Birth Cohort:	1925	1935	1945	1925	1935	1945	1925	1935	1945	1925	1935	1945	1925	1935	1945
Age:	Males														
25	47.1	47.0	46.9	49.6	50.8	52.0	50.4	51.8	53.2	53.1	55.8	58.5	54.4	57.8	61.2
45	29.5	29.4	29.3	31.7	32.7	33.8	32.3	33.6	34.9	34.8	37.2	39.7	35.9	39.0	42.3
65	14.6	14.5	14.4	16.2	17.0	17.8	16.7	17.7	18.7	18.6	20.5	22.6	19.5	22.0	24.8
85	5.4	5.3	5.3	6.2	6.6	7.1	6.5	7.0	7.6	7.5	8.7	10.1	8.1	9.7	11.7
Age:	Females														
25	54.7	53.0	51.3	57.7	57.6	57.6	58.6	58.9	59.3	60.5	61.9	63.3	61.0	62.8	64.7
45	35.9	34.4	32.9	38.7	38.6	38.6	39.6	39.9	40.1	41.3	42.6	44.0	41.8	43.5	45.3
65	19.0	17.8	16.6	21.3	21.2	21.2	22.0	22.3	22.5	23.5	24.6	25.8	23.9	25.4	27.0
85	7.1	6.5	5.8	8.5	8.5	8.5	9.0	9.2	9.3	10.0	10.8	11.6	10.3	11.4	12.6

Source: National Health Interview Survey-Linked Mortality File, 1986-2006
Note: Adjusted for race/ethnicity
